# Salvage radiotherapy for biochemical relapse after complete PSA response following radical prostatectomy: outcome and prognostic factors for patients who have never received hormonal therapy

**DOI:** 10.1186/1748-717X-2-8

**Published:** 2007-02-22

**Authors:** Alexandre A Jacinto, Angelo BS Fede, Lívia A Fagundes, João V Salvajoli, Marcus S Castilho, Gustavo A Viani, Ricardo C Fogaroli, Paulo ERS Novaes, Antonio Cássio A Pellizzon, Maria AC Maia, Robson Ferrigno

**Affiliations:** 1 Department of Radiation Oncology, Hospital do Cancer A C Camargo, São Paulo, Brazil

## Abstract

**Objectives:**

To evaluate the results of salvage conformal radiation therapy (3DC-EBRT) for patients submitted to radical prostatectomy (RP) who have achieved complete PSA response and who have never been treated with hormonal therapy (HT).

To present the results of biochemical control, a period free from hormonal therapy and factors related to its prognosis.

**Materials and methods:**

from August 2002 to December 2004, 43 prostate cancer patients submitted to RP presented biochemical failure after achieving a PSA < 0.2 ng/ml. They have never received HT and were submitted to salvage 3DC-EBRT. Median age was 62 years, median preoperative PSA was 8.8 ng/ml, median Gleason Score was 7. Any PSA rise above 0.2 was defined as biochemical failure after surgery. Median 3DC-EBRT dose was 70 Gy, biochemical failure after EBRT was defined as 3 consecutive rises in PSA or a single rise enough to trigger HT.

**Results:**

3-year biochemical non-evidence of disease (BNED) was 71%. PSA doubling time lower than 4 months (p = 0.01) and time from recurrence to salvage EBRT (p = 0.04) were associated with worse chance of biochemical control. Biochemical control of 76% was achieved when RT had been introduced with a PSA lower than 1 ng/ml vs. 48% with a PSA higher than 1 (p = 0.19). Late toxicity was acceptable.

**Conclusion:**

70% of biochemical control in 3 years can be achieved with salvage radiotherapy in selected patients. The importance of PSADT was confirmed in this study and radiotherapy should be started as early as possible. Longer follow up is necessary, but it is possible to conclude that a long interval free from hormonal therapy was achieved with low rate of toxicity avoiding or at least delaying several important adverse effects related to hormonal treatment.

## Background

Radical prostatectomy (RP) is an efficient method of achieving prostate cancer control. The follow-up is based on clinical history, physical exams and following the Prostate Specific Antigen (PSA) kinetics. Of the patients who fail to achieve biochemical control, the principle of selecting the best salvage therapy is based upon determining whether the disease is still on the prostate bed or if it has already spread throughout the body. Several studies are aiming at determining which variables correlate with a higher chance of detecting localized recurrences. In these cases, adopt a potentially curative salvage therapy (radiotherapy) instead of hormonal therapy alone.

The problem with many of these studies is that they usually include a broad group of patients who have never reached an indetectable PSA or those who have been previously treated with some sort of hormonal therapy making assessment using PSA difficult to evaluate.

The objective of this study is to evaluate the results of biochemical control and to analyze a period free from hormonal therapy after salvage radiation therapy in a selected group of patients who have never been treated before with hormonal therapy and have achieved complete PSA response after RP.

The secondary objectives are to evaluate prognostic factors related to the success of the salvage radiation therapy.

## Materials and methods

### Patients

From August 2002 to December 2004, 79 prostate cancer patients previously treated with radical prostatectomy (RP) were submitted to salvage three-dimensional conformal external beam radiation therapy (3DC-EBRT) due to biochemical failure. Thirty-six patients were excluded from the analysis: those who have not achieved PSA nadir (<0.2 ng/ml) after RP or those who were submitted to hormonal therapy before or during salvage radiation. Forty three patients were eligible. The median age was 62 years (range 50–73) and Caucasians were predominant (88.4%). Patients and treatment characteristics are show in Table 1.

**Table 1:** Patients' characteristics

		**N**	**%**
**Clinical Stage:**			
	T1c	6	14
	T2a	3	7
	T2b	2	4.7
	T2c	1	2.3
	Unknown	31	72

**Preoperative PSA:**			

	<10 ng/ml	24	55.80
	10–20 ng/ml	9	21.0
	>20	8	18.60
	Unknown	2	4.6

**Preoperative Gleason Score:**			

	<6	10	23.2
	.6	12	27.9
	>6	6	13.9
	unknown	15	34.9

**Pathological Stage:**			

	pT2a	11	25.6
	pT2b	3	7
	pT2c	7	16.3
	pT3a	21	48.8
	pT3b	1	2.3

**Postoperative Gleason Score:**			

	<6	7	16.3
	.6	13	30.2
	>6	23	53.5

**Surgical margins:**			

	Compromised	23	53.5
	Clear	20	46.5

**PNI:**			

	Yes	30	69.7
	No	6	14
	Unknown	7	16.3

**LVI:**			

	Yes	6	14
	No	25	58
	Unknown	12	28

**BVI:**			

	Yes	3	7
	No	27	62.8
	Unknown	13	30.2

**PIN:**			

	Yes	11	25.6
	No	7	16.3
	Unknown	25	58

**Total:**		43	100

PNI, perineural invasion; LVI, Limphovascular space invasion,

BVI, blood vascular space invasion; PIN, prostatic intraepithelial neoplasia

### Preoperative characteristics

Six patients (14%) were cT1c, 3 (7%) were cT2a, 2 (4.7%) were cT2b, 1 (2.3%) was cT2c. In 31 patients (72.1%) preoperative staging was not available. Median pre-operative PSA was 8.8 ng/ml (range 3 – 62) and hormonal therapy was not administered to any of then prior to surgery. Twenty-eight patients (65%) had information on the biopsy specimen, the median Gleason score was 6 (range 4 – 8).

### Biochemical recurrence

We defined biochemical recurrence after surgery as a single PSA value greater than 0.2 ng/ml after surgery in men with no evidence of distant metastasis at the time of radiotherapy.

After salvage radiotherapy, biochemical failure was based on the ASTRO (American Society of Therapeutic Radiation Oncology) criteria as three consecutive PSA rises or a single rise was high enough to trigger the initiation of hormone therapy.

### Postoperative characteristics

In surgical staging according to 2002 AJCC staging system, 11 patients (25.6%) were pT2a; 3 patients (7%) were pT2b; 7 (16.3%) were pT2c; 21 (48.8%) were pT3a and only 1 patient (2.3%) was pT3b. The median Gleason score was 7 (range 4 – 8). Surgical margin was affected in 23 patients (53.5%). Perineural invasion (PNI) was found in 30 patients (69.8%); There was no information regarding PNI, Lymphatic invasion (LI), Vascular invasion (VI) and intraepithelial neoplasia (PIN) in respectively 16.3%, 28%, 30.2% and 58% (Table 1).

After surgery the median interval to failure was 12 months (range 2 – 39). Median PSA before salvage radiotherapy was 0.87 ng/ml (range 0.24 – 7.9). PSA doubling time (PSADT) was calculated for each patient based on logarithmic regression formula and we used at least 2 PSA values separated by 2 months in the 18 months before salvage radiation.

At the time of recurrence all patients were submitted to physical examination which included a digital rectal exam, chest radiography, whole body bone scan and a trans-rectal pelvic ultrasonography. Eleven patients (25.6%) presented a nodule in the prostatic bed.

### Radiotherapy

Patients were submitted to external beam radiation therapy with a 10 MV linear accelerator (CLINAC 2100 – Varian^®^) using conformal three-dimensional technique. All patients were submitted to a pre-planning set up in a simulator (Acuity – Varian^®^) with retrograde urethrogram to help define the isocenter. A pelvic computed tomography (CT) was then performed to delinement of planning target volume. Twenty patients (46.6%) were treated with 2 planning tumor volumes (PTV): PTV1 included the surgical prostate bed and seminal vesicles bed with margins and the PVT2 included only the surgical prostate bed with margin. This technique was used according to the attending physician preferences based on post-surgical pathological information and pre treatment prostate image characteristics. Nineteen out of 20 patients treated this way had T3 tumors. Median dose to PTV1 was 50.4 Gy (range 46 – 54) and for all patients median dose to the prostate bed was 70 Gy (range 66 – 72). Median dose per fraction was 2 Gy (range 1.8 – 2).

### Follow-up

PSA was ordered every 3 months in the first year, every 4 months in the second year and yearly thereafter. Digital rectal examination was performed twice yearly.

### Statistical Analyses

Variables were evaluated using the chi-square test. Kaplan-Meier test was used to calculate overall and specific survival. Univariate analysis was assessed using the log-rank-test and Multivariate analysis was performed by Cox regression.

### Morbidity

Complications were recorded for genitourinary and gastrointestinal side effects. All acute and late complications were scored according to the Radiation Therapy Oncology Group (RTOG) scale.

## Results

**Figure 1:**
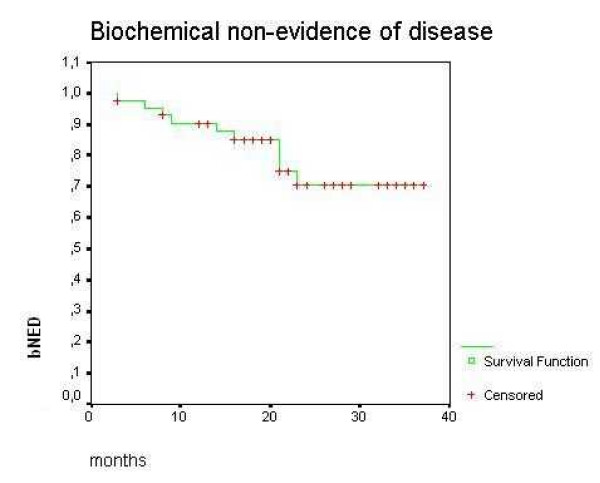
Biochemical control after salvage radiotherapy for patients who have never received hormonal therapy after radical prostatectomy.

Median interval from RP to biochemical failure was 12 months (range 2 – 39) and median time after failure to salvage 3DC-EBRT was 8 months (range 1 – 52). The median follow-up after radiotherapy was 26 months (range 8 – 41). One (1) patient (2.3%) was lost to follow-up. At the end of data collection no patients had died. Distant metastasis developed in 2 (4.7%) patients and 33 patients (76.7%) were free from biochemical failure. Of these, 28 patients (85%) developed undetectable PSA (<0.1 ng/ml) after a median interval of 3 months (range 1 – 30). Actuarial Biochemical Non-Evidence of Disease (BNED) at 3 years was 70.71% (Figure 1).

Median PSA pre-radiation was 0.87 ng/ml (range 0.21 – 7.9) and median PSADT was 5.25 months (range 1.0 – 16.5). Univariate and multivariate analysis of selected variables are displayed on Table 2.

**Table 2:** Uni and multivariate analysis

		**3-year BNED (%)**	**Univariate analysis**	**Multivariate analysis**
	**Variable**		***p***	***p (95% CI)***

**Gleason Score**				
	≤7	61	0.25	0.38
	>7	78		

**Pathological Stage**				

	≤pT3a	77	0.59	0.63
	pT3a or pT3b	64		

**Surgical Margins**				

	compromised	67	0.65	0.5
	clear	74		

**PNLVSI**				

	positive	68	0.31	0.29
	negative	100		

**Time to recurrence after RP**				

	≤12 meses	75	0.9	0.85
	>12 meses	66		

**PSADT**				

	≤4 meses	48.4	0.01	0.01
	>4 meses	75.6		

**Preradiation PSA**				

	≤1 ng/ml	75.9	0.1	0.7
	>1 ng/ml	48.1		

**Time to radiation after recurrence**				

	≤3 months	100	0.04	0.1
	>3 months	60		

**Clinical tumor on surgical bed**				

	Yes	80	0.4	0.46
	No	66		

**Radiation dose (Gy)**				

	≤66 Gy	76	0.62	0.7
	>66 Gy	68		

CI, confidence interval; PNLVSI, perineural or lymphatic or vascular space invasion; PSADT, PSA doubling time; Gy, Gray.

**Figure 2:**
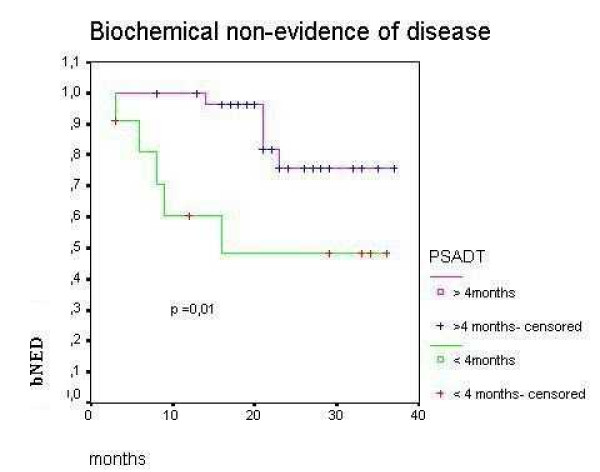
Biochemical control after salvage radiotherapy according to PSADT (PSA doubling time) lower or higher than 4 months for patients who have never received hormonal therapy after radical prostatectomy.

**Figure 3:**
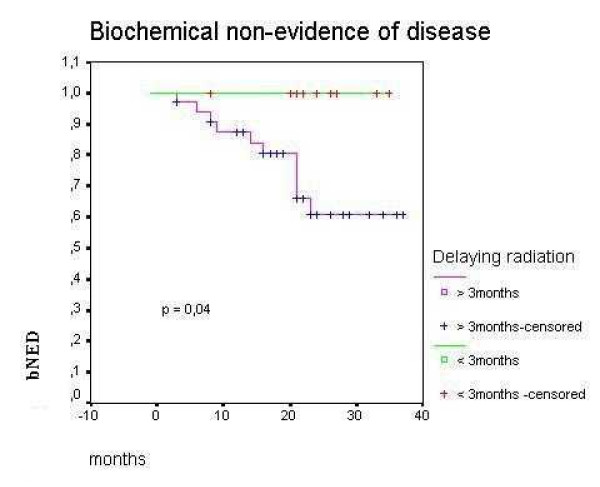
Biochemical control after salvage radiotherapy according to time to radiation after biochemical recurrence for patients who have never received hormonal therapy after radical prostatectomy.

PSADT lower than 4 months was an important negative prognostic factor to BNED/3-years (48.4% vs 75.6%, p = 0.012 – Figure 2). Delaying radiation therapy after biochemical recurrence for more than 3 months was associated with worse BNED/3-years (60% vs 100%, p = 0.04 – Figure 3). BNED/3-years for patients with PSA pre radiation higher than 1 ng/ml was 48.13% versus 75.9% for patients with lower PSA levels before radiation although it did not reach statistical significance (p = 0.19). Patients with clinical or radiological evidence of a macroscopic tumor on surgical bed showed BNED/3-years of 80.81% versus 66.5% for patients without evidence of local disease. Again, it did not reach statistic significance (p= 0.44).

Interval from surgery to biochemical failure, Gleason score, extracapsular extension, lymphovascular invasion, perineural extension or surgical margin involvement were not associated with prognosis.

BNED/3-years for patients submitted to radiotherapy including seminal vesicle bed was 81.64% versus 61.5% for patients submitted to radiotherapy to prostatic bed only without reaching statistical significance (p = 0.2). Total dose to surgical bed higher than 66 Gy did not result in better BNED/3-years (p = 0.6).

By multivariate analysis only a PSADT lower than 4 months was a negative predictive factor for BNED/3-years (p = 0.01; CI 95% – Table 3).

**Table 3:** Crude incidence of gastrointestinal and genitourinary morbidity

**Toxicity**	**Grade 1**	**Grade 2**	**Grade 3**	**Grade 4**
**Gastrointestinal**				

Acute	8(19.4%)	4(10%)	1(2.3%)	0
Late	2(5%)	4(10%)	1(2.3%)	0

**Genitourinary**				

Acute	6(15%)	4(10%)	1(2.3%)	0
Late	4(10%)	1(2.3%)	6(14.6%)	0

According to the RTOG morbidity scale 6 patients presented grade 3 late genitourinary effects (14.6%) and 1 patient (2.3%) presented grade 3 late gastrointestinal toxicity. No grade 4, acute or chronic, toxicity was seen. Table 3 shows the crude incidence of gastrointestinal and genitourinary complications. Grade 3 genitourinary morbidity was higher for patients who received radiotherapy for seminal vesicle bed, but without statistical significance (21 vs 8%, p = 0.38).

## Discussion

Prostate cancer is an indolent disease and the best way to evaluate disease control after radical treatment is monitoring PSA. It is estimated that about 1/3 of patients with biochemical failure following radical treatment will develop distant metastasis in a period of 8 years [1], but it is not a consensus whether or not biochemical control will improve survival [2].

After RP 70% of patients will achieve biochemical control in 10 years [3-6]. However, the appropriated definition of biochemical failure after surgery and PSA failure have been defined by different authors as a PSA greater than 0.2, 0.3, 0.4 or 0.5 ng/ml after RP [1,7-9]. Only patients with undetectable PSA after RP were included in the present study and we defined biochemical failure after surgery as any PSA value higher than 0.2 ng/ml.

Several variables have been described as prognostic factors for failure after surgery: histological grade (Gleason score); capsular or seminal vesicles extension; positive lymph nodes and involvement of surgical margins [10-12]. In our analysis 51% of patients had capsular involvement; Gleason score higher than 6 was found in 53.5% and margins were affected in 53.5%. No pathological characteristics were related to biochemical control probably due the small number of patients and short follow up of this cohort. However it is important to emphasize that our data differ from other studies since we have selected only patients who had indetectable PSA after surgery and who had never been submitted to hormonal treatment.

Salvage radiotherapy after RP is the only potential curative modality, but several published series have not demonstrated uniform results of BNED (18 to 68%) [9,13-27].

The most important issue in patients with biochemical failure is to define which patients will benefit from salvage treatment to the prostate bed. Unfortunately, an increase in PSA level after local treatment does not distinguish local recurrence from distant metastasis. Usual image exams or biopsy have not proved yet to be helpful in defining anatomical site of biochemical recurrence [21,28,29]. This fact could then explain the unfavorable outcomes with salvage radiation described in several series specially early published series [30]. Radioimmunoscintigraphy (RIS) is a new exam that could be helpful in defining this issue as recently suggested by Jani. They have presented promising results from a study with RIS and it is possible that the RIS could impact decision making [31].

In our experience BNED in 3 years was 71%, a result that is compatible with results from other institutions [9,16,22].

Although the follow-up is somewhat short for accurately defining the effects of the salvage therapy on local control or survival, another important result of the salvage radiation treatment that should be considered is the effect on the quality of life by delaying hormonal therapy. Hormonal therapy has been shown to produce deleterious side effects on the bone mass inducing higher chance of fractures on the spine and bones that carry the body weight [32].

The results of the present series of 70% BNED in 3 years will probably reflect on the patients' quality of life although we did not raise data to support such a conclusion.

Lately, several published series have pointed out adverse factors that could define patients with lower probability of occult distant metastasis which might result in better patient selection for local salvage treatment. The worst prognostic factors related to salvage radiation up to this moment are: higher Gleason score [9,15,16,18,24-26]; capsular or seminal vesicles extension [9,18,19,22,25,33]; free-surgical margins [9,33]; short PSADT [9,20,34-37] and high preradiotherapy PSA level [13,15,16,18,20-22,26,29]. Stephenson et al published recent results of the largest retrospective series which pooled 501 patients of 5 institutions. Results from this study have confirmed the negative prognostic value of high Gleason score (>7), pathological staging (pT3b), short PSADT (<10 months) and preradiation PSA level (>2 ng/ml) [9]. In our analysis PSADT < 4 months was strongly correlated with lower biochemical control (p = 0.01) reinforcing the importance of PSA kinetics to the outcome. Gleason score, vascular invasion, capsular extension and free surgical margin were not related to BNED in the present study probably due to the relatively small number of patients and the short follow-up.

In a recent prospective trial from the European Organization for Research and Treatment of Cancer (EORTC 22911), Bolla et al published results of a randomized comparison of wait-and-see after RP or immediate postoperative radiotherapy for high risk patients (pT3a, pT3b or positive margin) and has shown that adjuvant radiotherapy results in better progression free and local-regional free survival [8]. In small retrospective series [13,15,16,18,20-22,26] and in the series published by Stephenson et al [9], patients with low preradiotherapy PSA levels had better prognosis than those with high PSA. Therefore the outcome is dictated by an earlier salvage treatment. The ASTRO (American Society of Therapeutic Radiation Oncology) consensus recommends salvage radiation only for patients with PSA lesser than 1.5 ng/ml [38]. In our experience BNED was better if patients were submitted to salvage radiation earlier than 3 months after biochemical recurrence (100 vs 60%, p = 0.04). The BNED was also better if patients were treated before achieving 1 ng/ml of PSA, however, without reaching significance (75 versus 48%, p = 0.1))

We performed salvage treatment using conformal three-dimensional radiotherapy with a median dose of 70 Gy (range 66 – 72 Gy) and we have not found correlation between radiation dose and BNED (p = 0.6). Small retrospective series suggest that conformal three-dimensional radiotherapy and doses higher than 64,8 Gy do correlate with better biochemical control [14,27], but it is definitely not a consensus and it was also not demonstrated by Stephenson et al (p = 0,24) [9]. ASTRO consensus suggests doses higher than 64 Gy for salvage radiation setting [38]. If adjuvant radiotherapy is used lower radiation doses may be sufficient [8].

There is no agreement regarding which volume should be treated in prostatectomized patients. In the EORTC 22911 radiotherapy was delivered using 2 planning target volumes. The first Planning Target Volume (PTV 1) was defined by the anatomical limits of the surgical bed including those of the seminal vesicles followed by a boost in a reduced PTV (PTV2) to the prostate bed [8]. In our experience similar treatment (with 2 PTVs) was employed in 46% of patients. There was no difference in biochemical control if patients were treated with 1 or 2 PTVs. This was probably due to the inclusion of the seminal vesicle bed in patients treated with only one PTV. Jani et al have demonstrated that the use of RIS to determine to probable relapsed tumor could increase the PTV if compared with CT based PTV and probably without great difference in toxicity, although more bladder volume could receive doses higher than 60 Gy [39,40].

Some series have described low rates of complications (RTOG > Grade 3) in patients submitted to salvage radiotherapy, but what is generally postulated is that toxicity is higher when radiotherapy is employed after surgery than with exclusive radiotherapy specially in the genitourinary tract [13,27]. Ascher et al have found only 3% of grade 3 or 4 late urinary toxicity [27]. Morris et al have described a 6% incidence of grade 3 urethral stenosis after salvage radiation and 5% after adjuvant radiotherapy [41]. Bolla et al have shown lower late toxicity grade in patients submitted to adjuvant radiation than in patients submitted to salvage radiation, although in adjuvant setting the dose employed was lower (60 Gy) [8]. With a median dose of 70 Gy we found grade 3 urinary toxicity in 6 patients (14%) and no late rectal toxicity. There was no difference in morbidity whether radiotherapy was specifically directed to seminal vesicle or if treatment was directed to surgical bed only (p = 0.38).

Our data suggest that approximately 70% of biochemical control in 3 years can be achieved with salvage radiotherapy in selected patients and that 66 Gy may be sufficient for disease control. The importance of PSADT was confirmed in our series and radiotherapy should be started as early as possible. Longer follow up is necessary to confirm these results, but at this moment it is possible to conclude that a long interval free from hormonal therapy was achieved with low rate of toxicity avoiding, or at least, delaying several important adverse effects related to hormonal treatment.

## References

[B1] Pound CR, Partin AW, Eisenberger MA, Chan DW, Pearson JD, Walsh PC (1999). Natural history of progression after PSA elevation following radical prostatectomy. JAMA.

[B2] Jhaveri FM, Zippe CD, Klein EA, Kupelian PA (1999). Biochemical failure does not predict overall survival after radical prostatectomy for localized prostate cancer: 10-year results. Urology.

[B3] Bianco FJ, Wood DP, Cher ML, Powell IJ, Souza JW, Pontes JE (2003). Ten-year survival after radical prostatectomy: specimen Gleason score is the predictor in organ-confined prostate cancer. Clin Prostate Cancer.

[B4] Hull GW, Rabbani F, Abbas F, Wheeler TM, Kattan MW, Scardino PT (2002). Cancer control with radical prostatectomy alone in 1,000 consecutive patients. J Urol.

[B5] Pound CR, Partin AW, Epstein JI, Walsh PC (1997). Prostate-specific antigen after anatomic radical retropubic prostatectomy. Patterns of recurrence and cancer control. Urol Clin North Am.

[B6] Zincke H, Oesterling JE, Blute ML, Bergstralh EJ, Myers RP, Barrett DM (1994). Long-term (15 years) results after radical prostatectomy for clinically localized (stage T2c or lower) prostate cancer. J Urol.

[B7] Amling CL, Bergstralh EJ, Blute ML, Slezak JM, Zincke H (2001). Defining prostate specific antigen progression after radical prostatectomy: what is the most appropriate cut point?. J Urol.

[B8] Bolla M, van Poppel H, Collette L, van Cangh P, Vekemans K, Da Pozzo L, de Reijke TM, Verbaeys A, Bosset JF, van Velthoven R, Marechal JM, Scalliet P, Haustermans K, Pierart M (2005). Postoperative radiotherapy after radical prostatectomy: a randomised controlled trial (EORTC trial 22911). Lancet.

[B9] Stephenson AJ, Shariat SF, Zelefsky MJ, Kattan MW, Butler EB, Teh BS, Klein EA, Kupelian PA, Roehrborn CG, Pistenmaa DA, Pacholke HD, Liauw SL, Katz MS, Leibel SA, Scardino PT, Slawin KM (2004). Salvage radiotherapy for recurrent prostate cancer after radical prostatectomy. JAMA.

[B10] Walsh PC, Partin AW, Epstein JI (1994). Cancer control and quality of life following anatomical radical retropubic prostatectomy: results at 10 years. J Urol.

[B11] Gerber GS, Thisted RA, Scardino PT, Frohmuller HG, Schroeder FH, Paulson DF, Middleton AW, Rukstalis DB, Smith JA, Schellhammer PF, Ohori M, Chodak GW (1996). Results of radical prostatectomy in men with clinically localized prostate cancer. JAMA.

[B12] Catalona WJ, Smith DS (1998). Cancer recurrence and survival rates after anatomic radical retropubic prostatectomy for prostate cancer: intermediate-term results. J Urol.

[B13] Wu JJ, King SC, Montana GS, McKinstry CA, Anscher MS (1995). The efficacy of postprostatectomy radiotherapy in patients with an isolated elevation of serum prostate-specific antigen. Int J Radiat Oncol Biol Phys.

[B14] Valicenti RK, Gomella LG, Ismail M, Mulholland SG, Strup S, Petersen RO, Corn BW, Lu JD (1998). Durable efficacy of early postoperative radiation therapy for high-risk pT3N0 prostate cancer: the importance of radiation dose. Urology.

[B15] Song DY, Thompson TL, Ramakrishnan V, Harrison R, Bhavsar N, Onaodowan O, DeWeese TL (2002). Salvage radiotherapy for rising or persistent PSA after radical prostatectomy. Urology.

[B16] Rogers R, Grossfeld GD, Roach M, Shinohara K, Presti JC, Carroll PR (1998). Radiation therapy for the management of biopsy proved local recurrence after radical prostatectomy. J Urol.

[B17] Ravery V, Lamotte F, Hennequin CH, Toublanc M, Boccon-Gibod L, Hermieu JF, Delmas V, Boccon-Gibod L (1998). Adjuvant radiation therapy for recurrent PSA after radical prostatectomy in T1-T2 prostate cancer. Prostate Cancer Prostatic Dis.

[B18] Pisansky TM, Kozelsky TF, Myers RP, Hillman DW, Blute ML, Buskirk SJ, Cheville JC, Ferrigni RG, Schild SE (2000). Radiotherapy for isolated serum prostate specific antigen elevation after prostatectomy for prostate cancer. J Urol.

[B19] Liauw SL, Webster WS, Pistenmaa DA, Roehrborn CG (2003). Salvage radiotherapy for biochemical failure of radical prostatectomy: a single-institution experience. Urology.

[B20] Leventis AK, Shariat SF, Kattan MW, Butler EB, Wheeler TM, Slawin KM (2001). Prediction of response to salvage radiation therapy in patients with prostate cancer recurrence after radical prostatectomy. J Clin Oncol.

[B21] Koppie TM, Grossfeld GD, Nudell DM, Weinberg VK, Carroll PR (2001). Is anastomotic biopsy necessary before radiotherapy after radical prostatectomy?. J Urol.

[B22] Forman JD, Duclos M, Shamsa F, Pontes EJ (1996). Predicting the need for adjuvant systemic therapy in patients receiving postprostatectomy irradiation. Urology.

[B23] Eulau SM, Tate DJ, Stamey TA, Bagshaw MA, Hancock SL (1998). Effect of combined transient androgen deprivation and irradiation following radical prostatectomy for prostatic cancer. Int J Radiat Oncol Biol Phys.

[B24] Chawla AK, Thakral HK, Zietman AL, Shipley WU (2002). Salvage radiotherapy after radical prostatectomy for prostate adenocarcinoma: analysis of efficacy and prognostic factors. Urology.

[B25] Cadeddu JA, Partin AW, DeWeese TL, Walsh PC (1998). Long-term results of radiation therapy for prostate cancer recurrence following radical prostatectomy. J Urol.

[B26] Brooks JP, Albert PS, Wilder RB, Gant DA, McLeod DG, Poggi MM (2005). Long-term salvage radiotherapy outcome after radical prostatectomy and relapse predictors. J Urol.

[B27] Anscher MS, Clough R, Dodge R (2000). Radiotherapy for a rising prostate-specific antigen after radical prostatectomy: the first 10 years. Int J Radiat Oncol Biol Phys.

[B28] Cher ML, Bianco FJ, Lam JS, Davis LP, Grignon DJ, Sakr WA, Banerjee M, Pontes JE, Wood DP (1998). Limited role of radionuclide bone scintigraphy in patients with prostate specific antigen elevations after radical prostatectomy. J Urol.

[B29] Thomas CT, Bradshaw PT, Pollock BH, Montie JE, Taylor JM, Thames HD, McLaughlin PW, DeBiose DA, Hussey DH, Wahl RL (2003). Indium-111-capromab pendetide radioimmunoscintigraphy and prognosis for durable biochemical response to salvage radiation therapy in men after failed prostatectomy. J Clin Oncol.

[B30] Lange PH, Lightner DJ, Medini E, Reddy PK, Vessella RL (1990). The effect of radiation therapy after radical prostatectomy in patients with elevated prostate specific antigen levels. J Urol.

[B31] Jani AB, Blend MJ, Hamilton R, Brendler C, Pelizzari C, Krauz L, Vijayakumar S, Sapra B, Awan A, Weichselbaum RR (2004). Influence of radioimmunoscintigraphy on postprostatectomy radiotherapy treatment decision making. J Nucl Med.

[B32] Shahinian VB, Kuo YF, Freeman JL, Goodwin JS (2005). Risk of fracture after androgen deprivation for prostate cancer. N Engl J Med.

[B33] Katz MS, Zelefsky MJ, Venkatraman ES, Fuks Z, Hummer A, Leibel SA (2003). Predictors of biochemical outcome with salvage conformal radiotherapy after radical prostatectomy for prostate cancer. J Clin Oncol.

[B34] Partin AW, Pearson JD, Landis PK, Carter HB, Pound CR, Clemens JQ, Epstein JI, Walsh PC (1994). Evaluation of serum prostate-specific antigen velocity after radical prostatectomy to distinguish local recurrence from distant metastases. Urology.

[B35] Patel A, Dorey F, Franklin J, deKernion JB (1997). Recurrence patterns after radical retropubic prostatectomy: clinical usefulness of prostate specific antigen doubling times and log slope prostate specific antigen. J Urol.

[B36] Roberts SG, Blute ML, Bergstralh EJ, Slezak JM, Zincke H (2001). PSA doubling time as a predictor of clinical progression after biochemical failure following radical prostatectomy for prostate cancer. Mayo Clin Proc.

[B37] Ward JF, Zincke H, Bergstralh EJ, Slezak JM, Blute ML (2004). Prostate specific antigen doubling time subsequent to radical prostatectomy as a prognosticator of outcome following salvage radiotherapy. J Urol.

[B38] Cox JD, Gallagher MJ, Hammond EH, Kaplan RS, Schellhammer PF (1999). Consensus statements on radiation therapy of prostate cancer: guidelines for prostate re-biopsy after radiation and for radiation therapy with rising prostate-specific antigen levels after radical prostatectomy. American Society for Therapeutic Radiology and Oncology Consensus Panel. J Clin Oncol.

[B39] Jani AB, Spelbring D, Hamilton R, Blend MJ, Pelizzari C, Brendler C, Krauz L, Vijayakumar S, Sapra B, Weichselbaum RR (2004). Impact of radioimmunoscintigraphy on definition of clinical target volume for radiotherapy after prostatectomy. J Nucl Med.

[B40] Jani AB, Blend MJ, Hamilton R, Brendler C, Pelizzari C, Krauz L, Sapra B, Vijayakumar S, Awan A, Weichselbaum RR (2004). Radioimmunoscintigraphy for postprostatectomy radiotherapy: analysis of toxicity and biochemical control. J Nucl Med.

[B41] Morris MM, Dallow KC, Zietman AL, Park J, Althausen A, Heney NM, Shipley WU (1997). Adjuvant and salvage irradiation following radical prostatectomy for prostate cancer. Int J Radiat Oncol Biol Phys.

